# Discovery of Selenocysteine as a Potential Nanomedicine Promotes Cartilage Regeneration With Enhanced Immune Response by Text Mining and Biomedical Databases

**DOI:** 10.3389/fphar.2020.01138

**Published:** 2020-07-24

**Authors:** Jing Ye, Bingbing Xu, Baoshi Fan, Jiying Zhang, Fuzhen Yuan, Yourong Chen, Zewen Sun, Xin Yan, Yifan Song, Shitang Song, Meng Yang, Jia-Kuo Yu

**Affiliations:** ^1^ Knee Surgery Department of the Institution of Sports Medicine, Peking University Third Hospital, Beijing Key Laboratory of Sports Injuries, Beijing, China; ^2^ School of Clinical Medicine, Weifang Medical University, Weifang, China

**Keywords:** cartilage, biomaterial drug, nanomedicine, immune response, clinical translation, text mining

## Abstract

**Background:**

Unlike bone tissue, little progress has been made regarding cartilage regeneration, and many challenges remain. Furthermore, the key roles of cartilage lesion caused by traumas, focal lesion, or articular overstress remain unclear. Traumatic injuries to the meniscus as well as its degeneration are important risk factors for long-term joint dysfunction, degenerative joint lesions, and knee osteoarthritis (OA) a chronic joint disease characterized by degeneration of articular cartilage and hyperosteogeny. Nearly 50% of the individuals with meniscus injuries develop OA over time. Due to the limited inherent self-repair capacity of cartilage lesion, the Biomaterial drug-nanomedicine is considered to be a promising alternative. Therefore, it is important to elucidate the gene potential regeneration mechanisms and discover novel precise medication, which are identified through this study to investigate their function and role in pathogenesis.

**Methods:**

We downloaded the mRNA microarray statistics GSE117999, involving paired cartilage lesion tissue samples from 12 OA patients and 12 patients from a control group. First, we analyzed these statistics to recognize the differentially expressed genes (DEGs). We then exposed the gene ontology (GO) annotation and the Kyoto Encyclopaedia of Genes and Genomes (KEGG) pathway enrichment analyses for these DEGs. Protein-protein interaction (PPI) networks were then constructed, from which we attained eight significant genes after a functional interaction analysis. Finally, we identified a potential nanomedicine attained from this assay set, using a wide range of inhibitor information archived in the Search Tool for the Retrieval of Interacting Genes (STRING) database.

**Results:**

Sixty-six DEGs were identified with our standards for meaning (adjusted P-value < 0.01, |log2 - FC| ≥1.2). Furthermore, we identified eight hub genes and one potential nanomedicine - Selenocysteine based on these integrative data.

**Conclusion:**

We identified eight hub genes that could work as prospective biomarkers for the diagnostic and biomaterial drug treatment of cartilage lesion, involving the novel genes *CAMP*, *DEFA3*, *TOLLIP*, *HLA-DQA2*, *SLC38A6*, *SLC3A1*, *FAM20A*, and *ANO8*. Meanwhile, these genes were mainly associated with immune response, immune mediator induction, and cell chemotaxis. Significant support is provided for obtaining a series of novel gene targets, and we identify potential mechanisms for cartilage regeneration and final nanomedicine immunotherapy in regenerative medicine.

## Introduction

Cartilage lesion occur as a result of destructive joint diseases, such as osteoarthritis (OA) (Hu et al., 2018). They can cause disability, joint pain, movement limitation, and function impairment (Renders et al., 2014). Currently, there are no efficient actions for cartilage regeneration, due to the substandard inherent repair capacity of the damaged part of the cartilage lesion (Alcidi et al., 2007). Articular cartilage has the unique effect of conducting stress and reducing friction, and its damage can lead to joint dysfunction, and even disability (Gao et al., 2019). The articular cartilage itself has no blood supply, nerves, or lymphoid tissues. It also lacks chondrocytes, and has relatively low sculpting capacity (Cheng et al., 2019). The chondrocytes in the matrix pits have low metabolic activity (Lawrence et al., 2017). They mainly obtain necessary nutrients and excrete metabolites through penetration. The inability or difficulty to repair itself has been a major challenge for the orthopaedic community (Pang et al., 2013). Clinical treatments of articular cartilage lesion can be divided into being either reparative or non-reparative (Uehara et al., 2015). There are two types of reparative surgery: biological and non-biological. Non-reparative operations are debridement and joint irrigation. Non-biological methods, such as artificial joint prosthesis replacement and articular surface shaping, have achieved very good results and functional recovery (Yao et al., 2015; Ragni et al., 2019). Additionally, extreme and burning knee cartilage lesion often need total knee arthroplasty (TKA), which is a surgical option for easing the pain and facilitating knee reconstruction (Math et al., 2006).

The range of methods extensively used for cartilage regeneration in clinical practice involves distinctive types of scaffolds that imitate the native atmosphere, following: (1) mosaicplasty—the replacement of the missing cartilage with an autologous transplant-collected cartilage, such as the double layer collagen type I/III scaffold (MACI) (Liu et al., 2018); (2) microfracture—the disorder of the collagen scaffold, where double layer type I collagen sponge hols chondroitin sulfate to support recruitment of bone marrow stromal cells to the cartilage defect site (Wang et al., 2019); (3) ACI—the vitro culture of autologous chondrocytes that are collected from a special area of the cartilage and then injected into the defect site, covering them with a recyclable collagen membrane (Zhang et al., 2019); and (4) MACI—the transplantation of a viable scaffold surrounding the previous autologous chondrocytes culture (Li et al., 2016). However, currently, the effectiveness of these methods regarding cartilage regeneration remains far from satisfactory (Chen et al., 2020). Consequently, there is a vital need to identify potential mechanisms for cartilage regeneration, and to mine efficacious nanomedicines for regenerative medicine.

Bioinformatics is an emerging interdisciplinary field that manages the storing, repossession, sharing and best use of data and skills, for problem-solving and decision-making purposes (Hasman et al., 2011). The development and renewal of bioinformatics has provided us with the opportunity to mine large databases and uncover more meaningful solutions (Cheng, 2018). Massive databases have increased from cartilage lesion samples during these years, and a great deal of the differentially expressed genes (DEGs) have been determined using the gene ontology (GO) annotation and the Kyoto Encyclopaedia of Genes and Genomes (KEGG) pathway enrichment analyses (Huang et al., 2018). At present, some basic bioinformatics tools have been applied in the clinic, providing powerful weapons for clinical diagnosis, prevention, treatment, and clinical efficacy evaluation. For example, the GeneBank database and OMIM database are widely used by clinicians to search for pathogen or human disease-related gene information. They can then design specific primers and probes through biological software for the diagnosis, typing, quantification, and the identification of drug resistance genes of clinical genes. This plays an important role in the prevention of infectious diseases, genetic diseases, tumors, early diagnosis, treatment, and prognosis.

**Figure 1:**
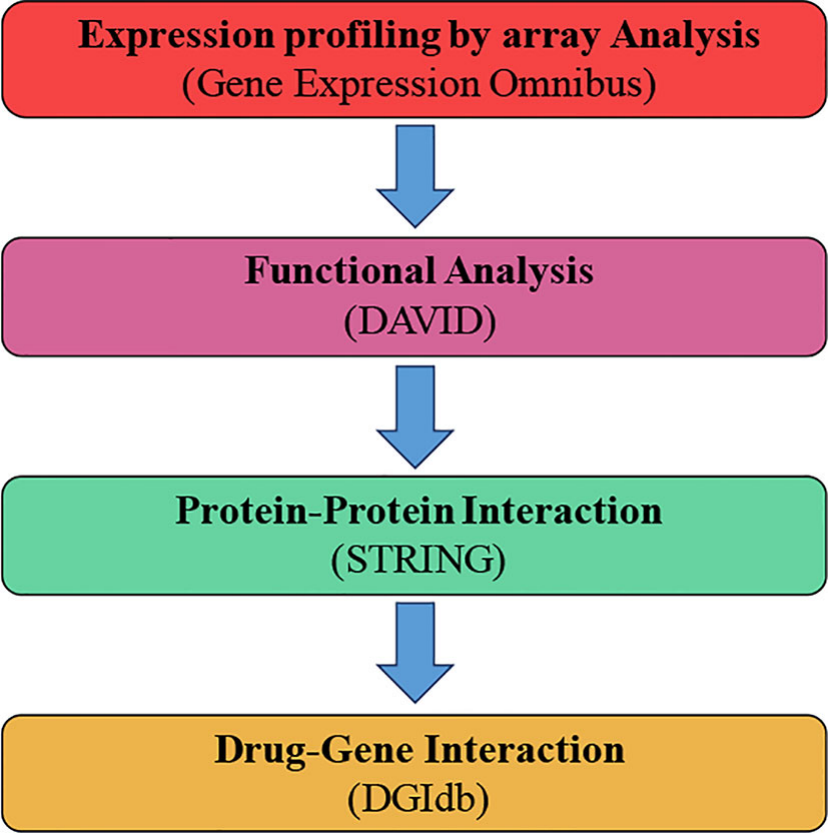
General text mining approach. The data mining was expanded to distinguish genes connected with osteoarthritis (OA) and patients without osteoarthritis (marked control) (N-OA), using the Gene Expression Omnibus. The obtained genes were investigated for their function and gene pathway using GO and KEGG. The enrichment was attained by PPI with STRING. The final enriched gene list was identified using the DGIdb.

**Figure 2:**
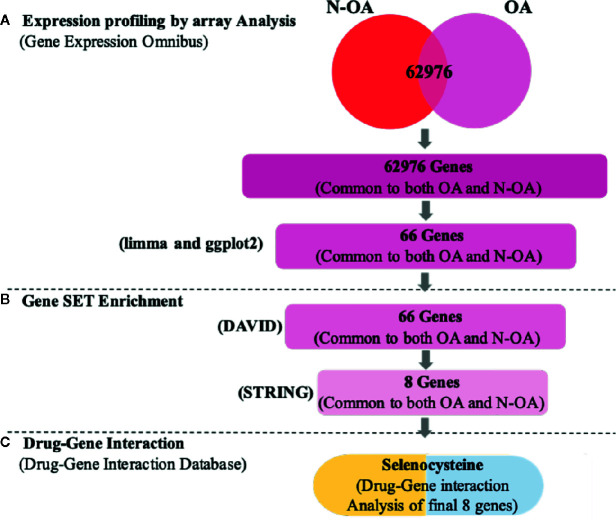
Work flow of statistics mining. **(A)** Text mining: on the pursuit concepts osteoarthritis (OA) and patients without osteoarthritis (N-OA), text digging was accomplished with GEO, and 62,976 genes were established. **(B)** Gene enrichment: gene enrichment analysis was accomplished with GO and KEGG to enrich 66 genes. Meanwhile, by uploading Search Tool for the STRING, 66 hub genes were enriched, and among them eight significant genes were targetable by drugs using DGIdb. **(C)** Drug-gene interaction: these eight genes were established in DGIdb and the inhibitor Selenocysteine drugs were recognized as having a prospective impact on cartilage regeneration.

In our analysis, we took the GSE117999 mRNA expression statistics with the Gene Expression Omnibus (GEO). DEG analyses were made from the cartilage tissue from 12 osteoarthritis patients and 12 patients without osteoarthritis (marked control), using the R software (Jalal et al., 2017) by means of the R-limma et al. packages (Ritchie et al., 2015). Subsequently, GO and KEGG analyses were accomplished using the website of DAVID (Database for Annotation, Visualization and Integrated Discovery). The Protein-protein interaction networks (PPI) networks were constructed, and eight HUB genes were identified. Finally, we discovered a cathelicidin antimicrobial peptide (*CAMP*) inhibitor, Selenocysteine, using the DGIdb database (Cotto et al., 2018). Using this approach (**Figure 1** **/** **Figure 2**), we recognized numerous potentially important cartilage regeneration - associated genes and pathways.

## Materials and Methods

### mRNA Microarray Statistics and Database Acquisition

The mRNA statistics text microarray GSE117999 (Rai MF et al., 2018) (the Patients/tissues details are given in the **Supplementary Material**) were download on the community GEO database (Barrett et al., 2013) (https://www.ncbi.nlm.nih.gov/geo), and executed on the GPL20844 platform. GSE117999 contains 12 patients with osteoarthritis, and 12 patients without osteoarthritis (arthroscopic partial meniscectomy). The normalized log2 ratio expressive OA/N-OA of the GSE117999 dataset, normalized by the L and Q process (Sbarrato et al., 2017), was taken. Analysis interested gene IDs were changed to gene symbols on the Agilent-072363 SurePrint G3 Human GE v3 8x60K Microarray 039494 (Feature Number Version). Institutional Review Board approved the study protocol. Prior to participation, a written informed consent was obtained from each patient (Brophy et al., 2018).

### DEG Analysis

DEG analysis is the genes expressed at meaningfully levels by numerous methods of analysis (Rahmatallah et al., 2016). We used limma and R to recognize the DEGs in the tissues of OA patients, and compared them to the patients without osteoarthritis (arthroscopic partial meniscectomy). Genes with |log2 FC| ≥1.2 and adjusted P values < 0.01 (moderated t-statistics, corrected by B and H method) were taken in the analysis period (Solari et al., 2017).

### GO and KEGG Analysis

GO (Carbon et al., 2017; Thomas, 2017) comprises a set of terms defining gene produces of biological process (BP), molecular function (MF), and cellular component (CC). KEGG (Kanehisa et al., 2017) offers statistics of recognized biological pathways. We used DAVID (Dennis et al., 2003) to envision the biological function and pathways enrichment of DEGs (The noteworthy was determined as p-value < 0.05).

### PPI Networks Construction

The STRING (Version 11.0) (Wei et al., 2019) catalogue was used. This databank includes over 25 million proteins and interactions, complexed within 5,000 organisms. The interaction score was set to ≥ 0.900, and the PPI (Protein-Protein Interaction) networks were created.

### Drug-Gene Interactions

Drugs were selected based on the hub genes that served as promising targets by using the Drug-Gene Interaction Database (DGIdb; http://www.dgidb.org/search_interactions). In this study, the final drug was approved by the Food and Drug Administration (FDA) (Yang et al., 2020). This study was prepared so that data on drug-gene interactions and gene target ability could be acquired. Furthermore, PubChem (https://www.ncbi.nlm.nih.gov/pccompound/) was used to confirm whether the medicines identified in our enquiry could target the genes that we identified.

### Quantitative Real-Time PCR (qRT-PCR)

After total RNA was extracted and mRNA purified (RNeasy Mini kit, Qiagen, Hilden, Germany), mRNA was converted to cDNA using the Trans-Scriptor First-Strand cDNA Synthesis SuperMix (TransScript, #AT301, Beijing, China). The assays-on-demand primers and probes and TaqMan Universal Master Mix were used to examine gene expression by the MiniOpticonTM RT-PCR system (Bio-Rad, Hercules, CA, USA) according to the user’s manual and amplified detection was conducted using SYBR-Green RealMastcrMix (Bio-Rad). The expression values of mRNAs were normalized against glyceraldehyde-3-phosphate dehydrogenase (GAPDH) and relatively calculated using 2^−ΔΔCt^ method (Ye et al., 2019).

## Results

### Identification of DEGs

**Figure 3:**
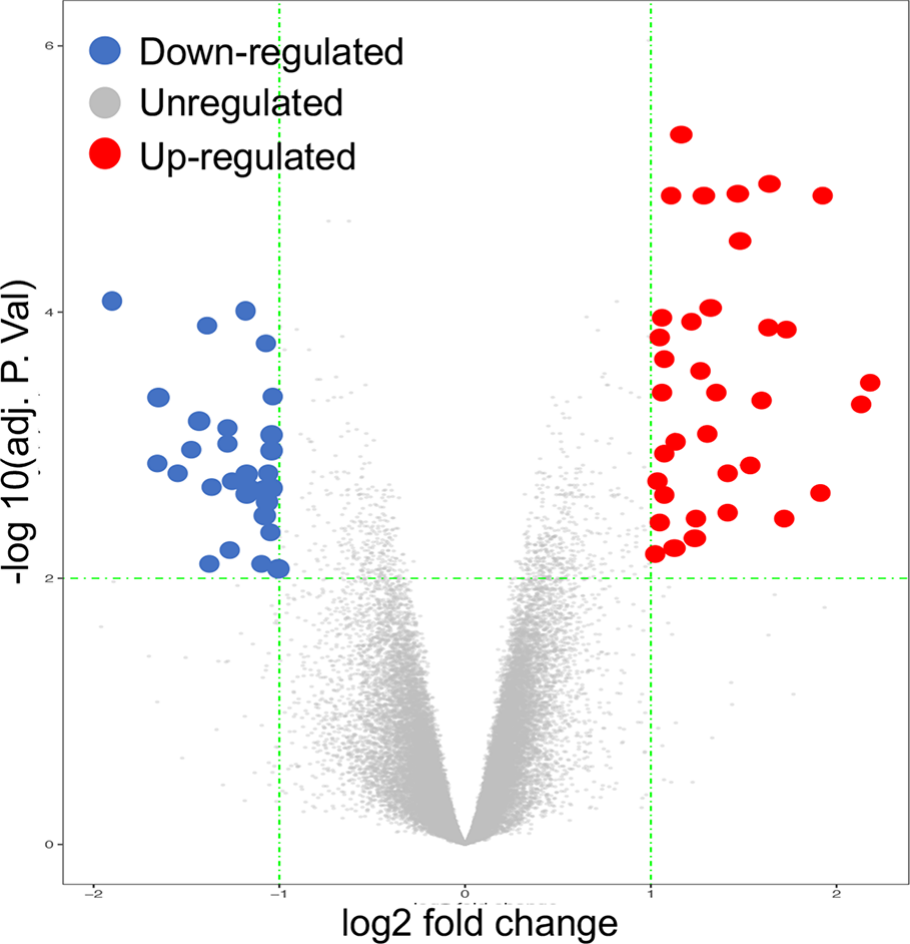
Volcano Plot image displaying the 66 genes that were significantly up-regulated or down-regulated (P-value < 0.05) in OA cartilage, compared to normal controls.

In our analysis, we recognized DEGs from 12 paired cartilage tissues (OA), using R-limma, compared with N-OA (**Figure 1** **/** **Figure 2**). Using both |log2 FC| ≥ 1.2 and adjusted p value < 0.01, 66 genes were recognized, including 35 UP-GENEs and 31 Down-GENEs (**Table 1**) (**Figure 3**) (**Supplementary Table**).

**Table 1:** Sixty-six DEG were recognized in GSE117999.

DEGs	Gene symbol
Upregulated Genes	*lnc-RP11-389E17.1.1-3, PHLDA3, lnc-AP1S2-2, THAP5*, *XLOC_l2_015590, lnc-NAIF1-1, lnc-PPAP2B-1, HOXC8*, *INCENP, LEO1, TSPAN11, PPP6R2, CRB1, ZNF354B*, *TRHDE-AS1, lnc-CTH-7, LCE2B, ESR1, QRFPR, SLC3A1, SMNDC1, LOC647323, CCR10, lnc-MKRN3-3*, *LOC102723716, ZSCAN12, DEFA3, FAM20A, FAM90A1*, *CAMP, ABCB5, LOC100506258, LINC00965, UHRF1BP1*, *PPIEL*
Downregulated genes	*MEG3, LOC101929767, HLA-DRB4, FRMD8, FNBP4*, *LOC100131564, PCDHB10, TOLLIP, HLA-DQA2*, *TMEM259, lnc-C9orf156-1, lnc-SPAG5-1, SLC38A6*, *lnc-AC233263.1-1, lnc-EIF2C2-2, ADHFE1, LINC00265*, *TP53TG3D, TRIM78P, FAM184B, SS18L1, CHST1*, *ANO8, lnc-TMED5-1, LOC100288069, DUT, SLC17A9*, *NRBP2, SLC25A37, COL27A1, NPIPB3*

The DEGs are sorted in adjusted p-value ascending order.

### GO and KEGG Analysis

**Figure 4:**
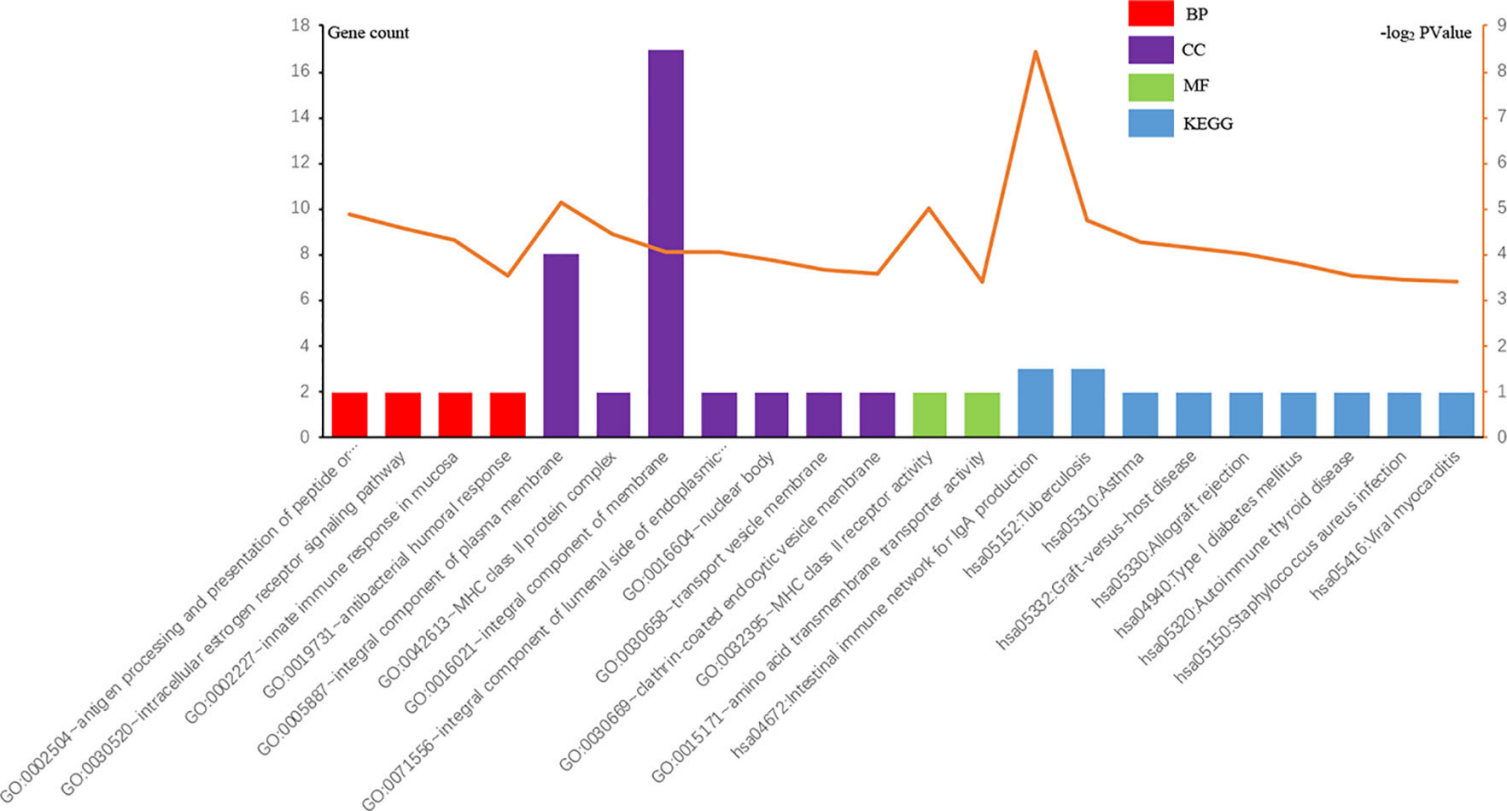
GO and KEGG pathways of DEGs meaningfully enriched in OA.

To discover further prospective targets of those DEGs in cartilage regeneration, we performed GO and KEGG analyses on OA, using a p value of <0.05 (**Figure 4**). As revealed in **Figure 4**, it demonstrations the all noteworthy terms for each of the following: the BP, CC, MF, and KEGG pathways of DEGs.

The marks of the UP-GENEs and the DOWN-GENEs are shown. As revealed in **Table 2**, in the BP group, the UP-GENEs were primarily enriched for genes complicated in the intracellular oestrogen receptor signalling pathway, innate immune response in mucosa and antibacterial humoral response. The DOWN-GENEs were enriched genes in the antigen processing and the presentation of peptide or polysaccharide antigen, *via* MHC class II. They were also enriched in antigen processing and presentation, and the interferon-gamma-mediated signalling pathway. In the CC group, the UP-GENEs were chiefly enriched for genes associated with the vital constituent of the plasma membrane. The DOWN-GENEs were enriched for genes associated with the MHC class II protein complex, the integral component of lumenal side of endoplasmic reticulum membrane, and nuclear bodies. In the MF group, the UP-GENEs were chiefly enriched from genes associated with transcription factor activity and sequence-specific DNA binding. The downregulated DEGs were enriched for genes with MHC class II receptor activity. In the KEGG pathway group, the DOWN-GENEs were enriched for genes in Asthma, Graft-versus-host disease, and Allograft rejection.

**Table 2:** The GO and KEGG Enrichment Analysis of DEGs.

Category	Term	Count	p Value
**Upregulated DEGs**			
**GOTERM_BP_DIRECT**	GO:0030520~intracellular estrogen receptor signaling pathway	**2**	**0.022284**
**GOTERM_BP_DIRECT**	GO:0002227~innate immune response in mucosa	**2**	**0.026475**
**GOTERM_BP_DIRECT**	GO:0019731~antibacterial humoral response	**2**	**0.046152**
**GOTERM_BP_DIRECT**	GO:0050830~defense response to Gram-positive bacterium	**2**	**0.087341**
**GOTERM_CC_DIRECT**	GO:0005887~integral component of plasma membrane	**5**	**0.054847**
**GOTERM_MF_DIRECT**	GO:0003700~transcription factor activity, sequence-specific DNA binding	**4**	**0.079278**
**Upregulated DEGs**			
**GOTERM_BP_DIRECT**	GO:0002504~antigen processing and presentation of peptide or polysaccharide antigen via MHC class II	**2**	**0.015085**
**GOTERM_BP_DIRECT**	GO:0019882~antigen processing and presentation	**2**	**0.048039**
**GOTERM_BP_DIRECT**	GO:0060333~interferon-gamma-mediated signaling pathway	**2**	**0.061605**
**GOTERM_BP_DIRECT**	GO:0031295~T cell costimulation	**2**	**0.067483**
**GOTERM_BP_DIRECT**	GO:0019886~antigen processing and presentation of exogenous peptide antigen via MHC class II	**2**	**0.079136**
**GOTERM_CC_DIRECT**	GO:0042613~MHC class II protein complex	**2**	**0.0227**
**GOTERM_CC_DIRECT**	GO:0071556~integral component of lumenal side of endoplasmic reticulum membrane	**2**	**0.02982**
**GOTERM_CC_DIRECT**	GO:0016604~nuclear body	**2**	**0.033867**
**GOTERM_CC_DIRECT**	GO:0030658~transport vesicle membrane	**2**	**0.038902**
**GOTERM_CC_DIRECT**	GO:0030669~clathrin-coated endocytic vesicle membrane	**2**	**0.041912**
**GOTERM_CC_DIRECT**	GO:0012507~ER to Golgi transport vesicle membrane	**2**	**0.05287**
**GOTERM_CC_DIRECT**	GO:0016021~integral component of membrane	**10**	**0.060694**
**GOTERM_CC_DIRECT**	GO:0030666~endocytic vesicle membrane	**2**	**0.066645**
**GOTERM_CC_DIRECT**	GO:0032588~trans-Golgi network membrane	**2**	**0.083116**
**GOTERM_MF_DIRECT**	GO:0032395~MHC class II receptor activity	**2**	**0.014129**
**KEGG_PATHWAY**	hsa05310: Asthma	**2**	**0.021622**
**KEGG_PATHWAY**	hsa05332: Graft-versus-host disease	**2**	**0.023763**
**KEGG_PATHWAY**	hsa05330: Allograft rejection	**2**	**0.026613**
**KEGG_PATHWAY**	hsa04940: Type I diabetes mellitus	**2**	**0.030165**
**KEGG_PATHWAY**	hsa04672: Intestinal immune network for IgA production	**2**	**0.033707**
**KEGG_PATHWAY**	hsa05320: Autoimmune thyroid disease	**2**	**0.037239**
**KEGG_PATHWAY**	hsa05150: Staphylococcus aureus infection	**2**	**0.038649**
**KEGG_PATHWAY**	hsa05416: Viral myocarditis	**2**	**0.040761**
**KEGG_PATHWAY**	hsa05321: Inflammatory bowel disease (IBD)	**2**	**0.045673**
**KEGG_PATHWAY**	hsa05140: Leishmaniosis	**2**	**0.050566**
**KEGG_PATHWAY**	hsa04612: Antigen processing and presentation	**2**	**0.054048**
**KEGG_PATHWAY**	hsa05323: Rheumatoid arthritis	**2**	**0.062364**
**KEGG_PATHWAY**	hsa05145: Toxoplasmosis	**2**	**0.077458**
**KEGG_PATHWAY**	hsa05169: Epstein-Barr virus infection	**2**	**0.085609**
**KEGG_PATHWAY**	hsa05322: Systemic lupus erythematosus	**2**	**0.093702**
**KEGG_PATHWAY**	hsa04514: Cell adhesion molecules (CAMs)	**2**	**0.099066**

### PPI Networks and Analysis

**Figure 5:**
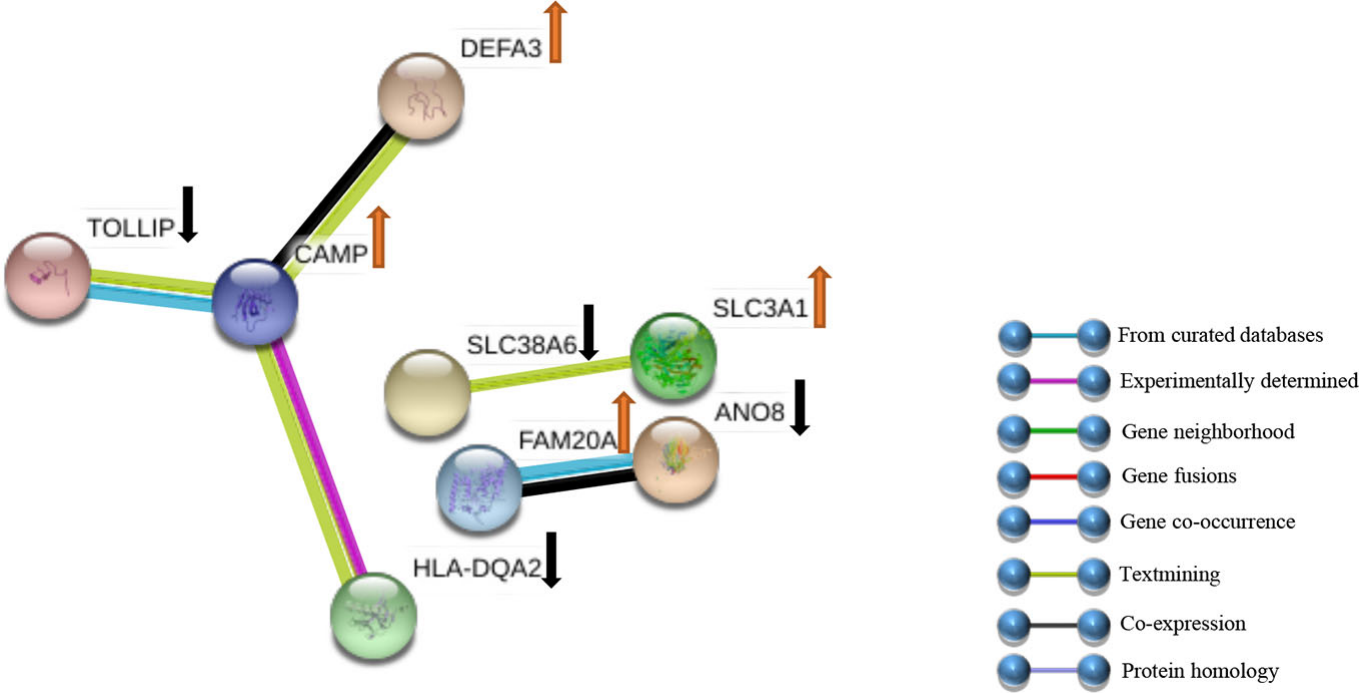
PPI networks of DEGs. Sixty-six genes were clarified into the PPI network using the STRING databank. ↑ indicates up-Genes; ↓ indicates down-Genes.

The DEGs were uploaded in STRING, and the results were explored. Eight HUB Genes with scores of >0.900 (maximum confidence) were chosen to create the PPI networks: *CAMP* and *TOLLIP*, *DEFA3*, *HLA-DQA2*, *SLC38A6*, *SLC3A1*, *FAM20A* and *ANO8* (**Figure 5**). *CAMP* translates the set of an antimicrobial peptide family, characterized by chemoattractant, immune mediator induction, and immunoreaction regulation (Snoussi et al., 2018; Uysal et al., 2019). Toll interacting protein (*TOLLIP*) encodes a ubiquitin-binding protein that regulates inflammatory signalling (Diao et al., 2016; Shah et al., 2017). Defensin alpha 3 (*DEFA3*) belongs to antimicrobial and cytotoxic peptides involved in host defence (Okazaki et al., 2007; Froy and Sthoeger, 2009). Major histocompatibility complex, class II, DQ alpha 2 (*HLA-DQA2*) are a family of the HLA class II alpha family. Many investigators suggest this involved in the release of CLIP molecule (Rudy and Lew, 1994). Solute carrier family 38 member 6 (*SLC38A6*) has possibly a relation with the glutamate-glutamine cycle regulation, responsible for preventing excitotoxicity (Schioth et al., 2013; Bagchi et al., 2014). Solute carrier family 3 member 1 (*SLC3A1*) translates a type II membrane glycoprotein that encodes neutral amino acids associated with cystinuria (Ma et al., 2018). FAM20A golgi is associated with the secretory pathway pseudokinase (*FAM20A*), which encodes a protein that might function in haematopoiesis and is associated with amelogenesis imperfecta and gingival hyperplasia syndrome (Beres et al., 2018; Koruyucu et al., 2018). Anoctamin 8 (*ANO8*) is associated with a human disorder that is often overexpressed in diverse cancers (Katoh and Katoh, 2005; Ousingsawat et al., 2011).

### Drug-Gene Interactions

**Figure 6:**
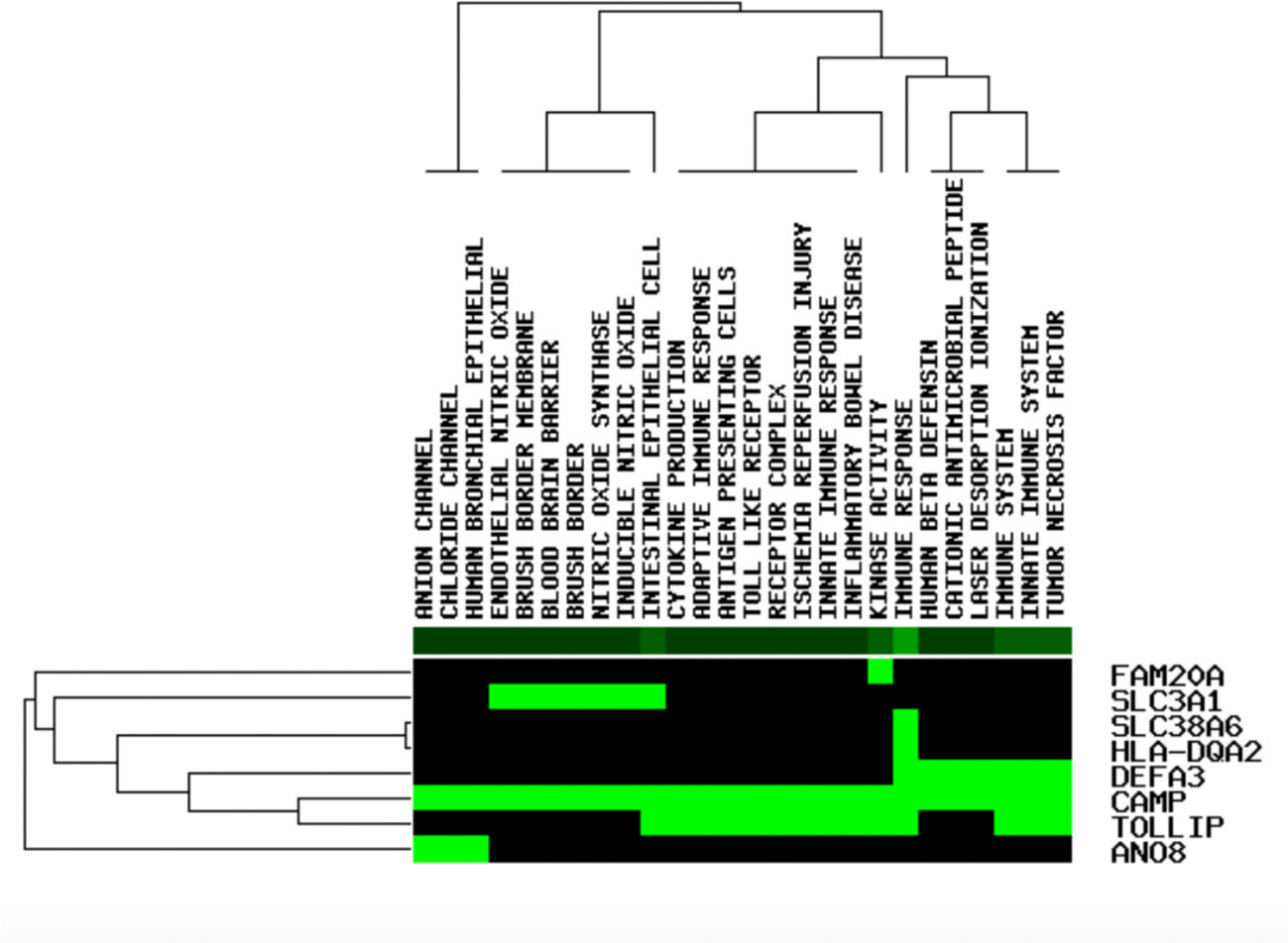
The functional enrichment analysis of eight applicant cartilage regeneration - associated genes (green shows that the gene is enhanced in the related pathway, black shows not on the contrary).

**Figure 7:**
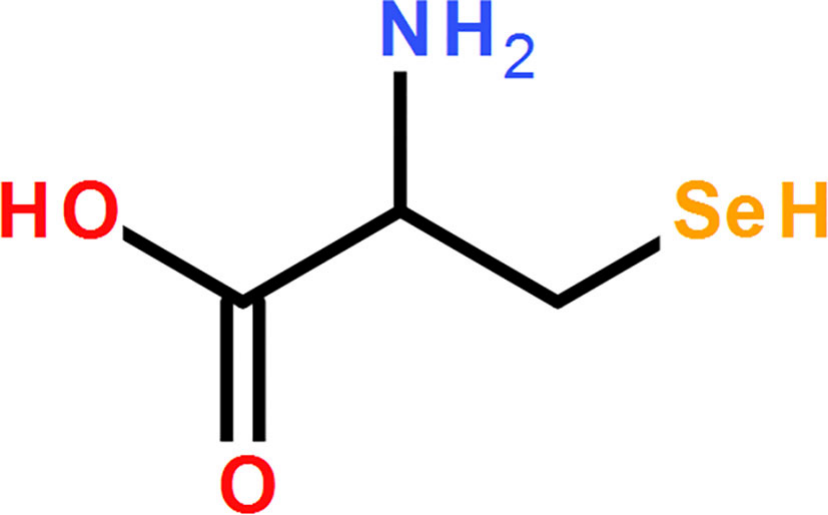
The schematic structure of Selenocysteine.

The functional enrichment investigation was conducted to identify the last prospective HUB genes with the sifter stricture, p value <1X10^10, as the edge in the GenCLiP 2.0 website (Wang et al., 2019) (**Figure 6**). DGIdb database was used to hunt the potential targets *CAMP* and its small organic compounds Selenocysteine. Selenocysteine is the main form of selenium in proteins. The study of selenocysteine biosynthesis and the mechanism of protein participation is a classic protein biochemical. This important supplement of molecular biology is also the basis for further research into the biological functions and applications of selenoproteins (Stadtman, 1996). The structure of Selenocysteine is shown in **Figure 7**.

### The Expression of *CAMP* was Inverse in OA Cartilage Tissues

**Figure 8:**
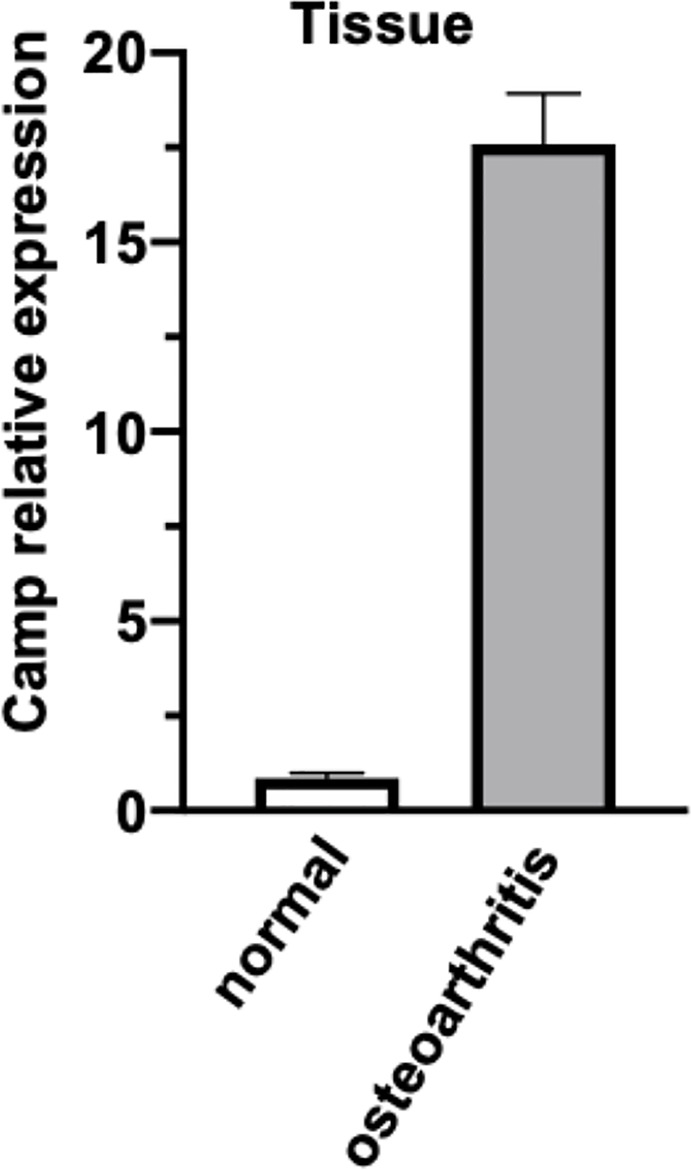
The expression of *CAMP* transcripts differentially expressed between normal and osteoarthritis was validated by real-time PCR. The data is presented as mean with standard error of the mean.

To examine the expression of mRNAs in the articular cartilage, qRT-PCR was performed in 3 samples. We found that the expression of *CAMP* was significantly upregulated in the OA group compared with the normal group (**Figure 8**).

## Discussion

This analysis was designed to recognize prospective cartilage regeneration - associated genes, by comparing cartilage tissues in patients without osteoarthritis (arthroscopic partial meniscectomy). Thirty-five UP and 31 DOWN DEGs were recognized. We accomplished GO and KEGG comment examinations. Next, a PPI network was created, and eight significant genes were recognized. Finally, eight genes, *CAMP* and *TOLLIP*, *DEFA3*, *HLA-DQA2*, *SLC38A6*, *SLC3A1*, *FAM20A*, and *ANO8* were identified as being significantly associated with immune response, immune mediator induction, and cell chemotaxis. The *CAMP* inhibitor Selenocysteine may be a nanomedicine potential candidate for cartilage regeneration.

In summary, only the *CAMP* gene has its commercial inhibitor of the eight hub genes, there have been many studies into the role of transcription factors in regulating the promoter region of the *CAMP* gene (Horibe et al., 2013). These studies have revealed that many biomolecules and factors can regulate the expression of the human antibacterial peptide *CAMP* gene, and that different signalling pathways can also affect the expression of the gene (Hancock and Diamond, 2000). Due to the important role that the *CAMP* protein plays in infectious diseases, tumors, and other diseases, it may be a target for the diagnosis and treatment of these diseases (Kovach et al., 2012). It is expected that the molecular regulatory mechanism of the *CAMP* gene during the occurrence of diseases, especially infectious diseases, will become a topic of significant further research (Rosenberger et al., 2004).

Selenocysteine is the main form of selenium in proteins. The determination of its codon UGA increases the number of amino acids that make up the biosynthetic protein, from 20 to 21 (Mousa et al., 2017). It is also the only amino acid that contains a metalloid element. It is mostly located in the active center of selenoprotein or selenoenzyme (especially in antioxidant enzymes). At the same time, as an essential trace element of the human body, it has a very significant role in anti-oxidation, immune regulation, and anti-tumor roles (Hatfield et al., 2014; Varlamova and Cheremushkina, 2017). This important supplement of molecular biology is also the basis for further research into the biological functions and applications of selenoproteins (Stadtman, 1996). It is one of the hotspots of research in the fields of protein biochemistry and molecular biology (Ren et al., 2018).

Interestingly, Se deficiency has been proposed as an underlying contributing factor for the chronic osteochondral disease Kashin-Beck disease, an important but neglected disease in parts of China. It was previously shown that Se(IV), as well as superoxide dismutase, can prevent damage done to cultured human embryonic cartilage cells caused by various etiological environmental substrates, and increase the activity of GSHpx while decreasing the production of lipid peroxides (Peng and Yang, 1991). In another report, the disease was associated with the incidence of Se deficiency in regions where the disease is prevalent (Peng et al., 1992). Finally, a mixture containing glycosaminoglycans, selenium, and vitamin E was shown to be exceptionally capable of promoting osteochondral repair in a rabbit model of knee osteochondral defect after 6 weeks of treatment (Handl et al., 2007). These past studies provide some evidence to support our current finding that selenocysteine may be effective drug to support cartilage regeneration.

To conclude, 66 prospective candidate cartilage regeneration - associated genes, which have earlier been involved in numerous pathways related to pathogenesis. All of these DEG applicant cartilage regeneration -associated genes should be further established through biological trials. Moreover, *CAMP*, *TOLLIP*, *DEFA3*, *HLA-DQA2*, *SLC38A6*, *SLC3A1*, *FAM20A*, and *ANO8*, as prospective markers for cartilage regeneration; they have not been linked previously, either in diagnosis or in research. Thus, the eight targets were considered to be potential therapeutic targets of cartilage regeneration. Thus, the *CAMP* inhibitor selenocysteine is considered to be a potential nanomedicine candidate for regenerative medicine.

## Data Availability Statement

The raw data supporting the conclusions of this article will be made available by the authors, without undue reservation.

## Author Contributions

All authors contributed to the data analysis. JYe and BX completed the analysis using bioinformatics instruments. All authors contributed to the article and approved the submitted version.

## Funding

This work was supported by the National Natural Science Foundation of China (ID. 5192010500) and the National Key R&D Program of China (No.2017YFB1303000).

## Conflict of Interest

The authors declare that the research was conducted in the absence of any commercial or financial relationships that could be construed as a potential conflict of interest.
