# Supplementary material for: Discovery of Selenocysteine as a Potential Nanomedicine Promotes Cartilage Regeneration With Enhanced Immune Response by Text Mining and Biomedical Databases
Source: Front Pharmacol. 2020 Jul 24;11:1138. doi: 10.3389/fphar.2020.01138 (PMC7394085; doi:10.3389/fphar.2020.01138)
Supplement: Supplementary file 1 [file Table_1.docx]

**About the Patients**

Institutional Review Board approved the study protocol. Prior to participation, a written informed consent was obtained from each patient. Patients of any age, body-mass-index (BMI) and from both sexes were included. Only those patients undergoing arthroscopic partial meniscectomy (APM) with no evidence for OA, cartilage chondrosis, bone-marrow lesions/edema, and no ligamentous injury > Grade-I medial-collateral ligament strain were included. Knees were assessed by radiographs using the Kellgren-Lawrence (K- L) scale for OA [1].

**About the Characteristics of study patients**

The study cohort included 12 patients without OA (K-L score=0) undergoing APM and 12 patients with OA (K-L score=3–4) undergoing TKA (Table-1). Age (P=0.0003) and BMI (P=0.0005) were significantly different between the two cohorts but the distribution by sex (75% female TKA cohort, 42% female APM cohort) was not(P=0.214). Condition (APM vs. TKA), age, BMI, and sex were included in the model as covariates [1].


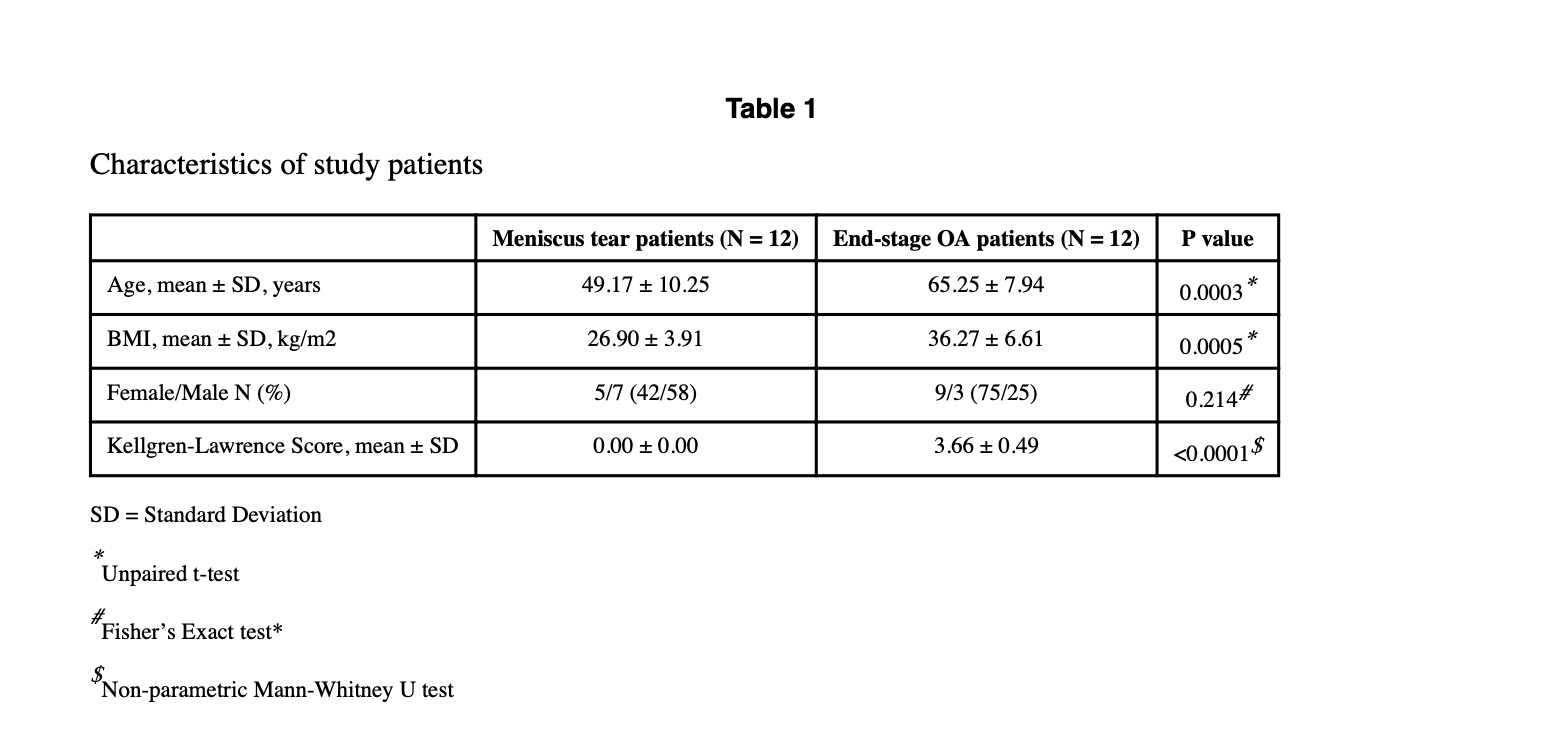


[1] R.H. Brophy, B. Zhang, L. Cai, R.W. Wright, L.J. Sandell, and M.F. Rai, Transcriptome comparison of meniscus from patients with and without osteoarthritis. Osteoarthritis Cartilage 26 (2018) 422-432.
